# Electrically stimulated gene expression under exogenously applied electric fields

**DOI:** 10.3389/fmolb.2023.1161191

**Published:** 2023-05-04

**Authors:** Sara Abasi, Abhishek Jain, John P. Cooke, Anthony Guiseppi-Elie

**Affiliations:** ^1^ Bioelectronics, Biosensors and Biochips (C3B), Department of Biomedical Engineering, Department of Electrical and Computer Engineering, Texas A&M University, College Station, TX, United States; ^2^ Department of Cardiovascular Sciences, Houston Methodist Institute for Academic Medicine and Houston Methodist Research Institute, Houston, TX, United States; ^3^ Department of Medical Physiology, College of Medicine, Texas A&M Health Science Center, Bryan, TX, United States; ^4^ Division of Engineering and Industrial Technology, Tri-County Technical College, Pendleton, SC, United States; ^5^ ABTECH Scientific, Inc., Richmond, VA, United States

**Keywords:** electromics, ECSARA, TEER, YAP, HUVEC, CD-144

## Abstract

**Introduction:** Electrical stimulation, the application of an electric field to cells and tissues grown in culture to accelerate growth and tight junction formation among endothelial cells, could be impactful in cardiovascular tissue engineering, allotransplantation, and wound healing.

**Methods:** Using Electrical Cell Stimulation And Recording Apparatus (ECSARA), the exploration of the stimulatory influences of electric fields of different magnitude and frequencies on growth and proliferation, trans endothelial electrical resistance (TEER) and gene expression of human endothelia cells (HUVECs) were explored.

**Results:** Within the range of endogenous electrical pulses studied, frequency was found to be more significant (*p* = 0.05) than voltage in influencing HUVEC gene expression. Localization of Yes Associated Protein (YAP) and expression of CD-144 are shown to be consistent with temporal manifestations of TEER.

**Discussion:** This work introduces the field of electromics, the study of cellular gene expression profiles and their implications under the influence of exogenously applied electric fields. Homology of electrobiology and mechanobiology suggests use of such exogenous cues in tissue and regenerative engineering.

## Introduction

Electric field (EF) biology, the influence of external electric fields on biological processes, or electrobiology, has long been a source of creative works (Nuccitelli, 1988; Yen-Patton et al., 1988; Hart and Palisano, 2017; Peng et al., 2021). Electric fields, voltage differentials, and membrane potentials are integral to cellular and tissue electrophysiology. Cellular electrical potentials play an important role in spatially delocalized, time-resolved signaling as observed in the nervous and cardiovascular systems. In these systems, cells generate action potentials, traveling waves of charged ion gradient changes, which alter membrane potentials and result in biochemical responses and differential gene expression (Fields et al., 1997; Ulrich et al., 2006). These electrical potentials are maintained by membrane-confined protein ion channels and active transporters which act to balance electrical and chemical potentials such that a specific voltage is maintained across the cell membrane (Wright, 2004; Benarroch and Asally, 2020). The membrane potential of cells, ranging between 0 and −100 mV, varies in proliferating *versus* non-proliferating cells pointing to a possible role of external fields on critical cellular performance during the cell cycle (Abdul Kadir et al., 2018). Such an intimate connection between cellular function and electric potentials suggests that cellular behavior may be modulated by application of an external EF. This endeavor is realized in the development of electric field biochemistry, oriented external-electric-fields (OEEF) that alter structure and chemical reactivity (Huang et al., 2019; Shaik et al., 2020), and electroceuticals, nascently-produced biochemicals whose levels may be altered with the use of an electric field (Majid, 2017) to influence cell and tissue behavior (Famm et al., 2013; Berggren et al., 2022). Applied electric fields may also induce physicochemical changes to transmembrane potentials, alter membrane permeability, or change the electroactivity of receptors or ligands (Abasi et al., 2020a; Stuyver et al., 2020). Electric fields have likewise been shown to influence the cytoskeleton and inhibit growth in cancer cell lines (Kirson et al., 2007). Indeed, there are several approved approaches already a mainstay in the clinic, such as cardiac pacemakers and antiarrhythmic devices (Gregoratos et al., 1998; Members et al., 2002). Here, a new term is defined, electromics, which is the study of cellular gene expression profiles and their implications under the influence of exogenously applied EFs. This field is a synthesis of bioelectronics and molecular biology, and this report describes preliminary work at this frontier through studies of EF-modulated YAP and CD144 expression biology in HUVECs.

EFs are normalized electrical forces per unit charge, with the Lorenz force ( $\vec{F}=q\vec{E}+q\vec{\upsilon }\;x\;\vec{B}$ ) describing the influence of an electric field $\vec{E}$ on a charged entity, q , moving at velocity, $\vec{\upsilon }$ , in a magnetic field, $\vec{B}$ . This relationship suggests that EFs apply forces throughout systems that contain charged species–a characteristic of all biological systems (Honig and Nicholls, 1995). This implies that electrostatic interactions and the resulting forces are a fundamental part of molecular biology beyond the action potential. These electrical forces are analogous with mechanical forces, such as shear stress or substrate stiffness, and thus may have convergent biology. Tangibly, ion channels and electrical potentials have been tied to molecular processes, outside of action potentials; ion channels in endothelial cells have been shown to be mechanosensitive and responsive to shear stresses (Lansman et al., 1987; Sachs and Morris, 1998), with examples including the recently elucidated piezo channels (Zhao et al., 2016; Moroni et al., 2018; Xiao, 2020). Electro-mechanical coupling in tissue maturation is well known for maturing cardiomyocytes from neonatal rats or from hiPSC differentiation; such maturation being facilitated by electrical or mechanical stimulation (Godier-Furnémont et al., 2015; Zhang et al., 2020). EFs have also been shown to influence gross cellular behavior in wound healing, influencing cell proliferation (Kamalov et al., 2022) and migration (Song et al., 2004; Zhao et al., 2004; Long et al., 2018). Taken together, this suggests a deep connection between the voltage potentials and forces induced by EFs, ion channel signaling, and the influence these may have on cellular genetics and biochemistry. Thus, there is considerable interest in harnessing the power of electric impulses to aid healing of chronic wounds, reduce pain, and restore neurological activity. However, fundamental understanding of the role of such electric pulses on cellular activity at the level of gene expression remains elusive.

Yes-associated protein 1 (YAP) and WW-domain-containing transcription regulator 1 (*transcriptional co-activator with PDZ-binding motif*) (TAZ) are the main effectors of the Hippo pathway, pivotal to tissue growth, and their nuclear localization is a well-established signature of the regulation of their transcriptional activity and role in signal transduction (Shreberk-Shaked and Oren, 2019). Such localization is well known to be influenced by external mechanical forces in pathways distinct from Hippo (Totaro et al., 2018; Kim et al., 2020), including shear stresses, such as in 3D printing of suspended cells or arising from flow over adherent cells (Panciera et al., 2017), and substrate stiffness (Dupont et al., 2011) or combinations of shear stress, flow regimen and substrate stiffness (Walther et al., 2021). Acting as transcriptional co-activators (Totaro et al., 2018), several genes are regulated by YAP/TAZ activity, for example connective tissue growth factor (CTGF) and ankyrin repeat domain 1 (ANKRD1) (Brusatin et al., 2018; Dupont, 2019). The downstream regulation of YAP/TAZ via several plasma membrane domains (Rausch and Hansen, 2020) suggests that membrane potentials and electrical influences that affect the plasma membrane (Lieu et al., 2004) may similarly and likewise affect YAP/TAZ localization.

Isolating such interactions between EFs and its downstream influence on cellular processes requires precise engineering of the interrogation system. To facilitate electromics, a cell culture system to culture and monitor the cellular response to uniform electrical fields in real time, termed the electrical cell stimulation and recording apparatus (ECSARA), was previously designed, fabricated, tested, and reported (Abasi et al., 2020a; Abasi et al., 2020b). The use of conventional 24-well cell culture plate format allows simultaneous, multiplexed investigations of gene expression under a uniform electric field orthogonal to the cell growth plane of trans-well cell culture inserts. In ECSARA, cells are kept away from direct contact with electrodes thus assuring isolation of the cells from interfacial effects (Abasi et al., 2020a). Additionally, ECSARA allows concomitant measurement of multiplexed, trans-well electrical impedance (Adler and Holder, 2021) to provide insight into endothelial tight junction formation (Cong and Kong, 2020). The engineered approach detailed here, applying ECSARA to study electrobiology, is of recent, general interest as controlling for the various physico-chemical parameters to study electrobiology is non-trivial (Brusatin et al., 2018). This report emphasizes the strengths of the engineered approach and demonstrates feasibility of assessing gene expression and YAP behavior under EF stimulation. Holistically, the engineered approach for studying electrobiology is of interest considering interfacial bioelectronics exert influences on cell behavior and devising methods to study the molecular biology is vital to understanding those underlying mechanisms.

**FIGURE 1:**
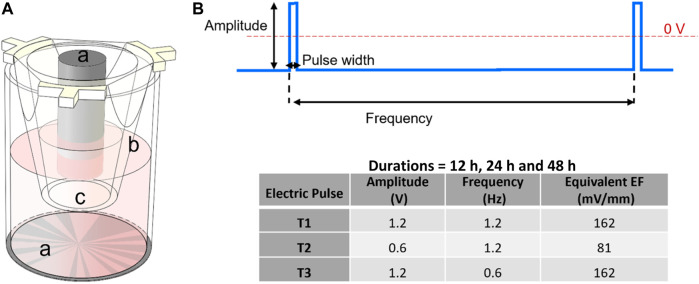
**(A)** Schematic of a cell culture insert within one well of the 24-well electroculture ware; labels shown are: a. Ti electrode pairs, b. cell culture insert, c. the insert’s nano-porous PET membrane. Cells were seeded and grown on this membrane. **(B)** Electrostimulation pulse: a pulse of 2 mS width was applied at frequencies of 1.2 and 0.6 Hz and amplitudes of 1.2 and 0.6 V for durations that were 12 h, 24 h and 48 h according to the inset table.

In this study, ECSARA was used in a novel application to specifically study YAP expression biology and CD144 expression under programmed EF influence. The effect of electrical stimulation on human umbilical endothelial cells (HUVECs) was studied by applying an EF under three electro-stimulation regimens alongside separately prepared, non-stimulated, negative controls for each regimen. ECSARA allowed parallel real-time monitoring of trans-endothelial electrical resistance (TEER; a resistance measure that assesses CD144 enabled tight junction formation between HUVECs) by multiplexed bioelectrical impedance spectroscopy (MBIS) and equivalent circuit analysis (EQCRTA). HUVECs were stimulated with programmed stand-off electric fields of 0 (non-stimulated, negative control), 2 mS pulses of amplitude 81 mV/mm (0.6 V) at 1.2 Hz (T1), 162 mV/mm (1.2 V) at 1.2 Hz (T2), and 162 mV/mm (1.2 V) at 0.6 Hz (T3) (Figure 1), where 1.2 Hz over a 48 h period. The frequency of 1.2 Hz was selected for its correspondence to the heart rate of 72 beats/min. The range of electric field strength was chosen to induce no temperature or pH change to the incubated (constant 37°C) and buffered cell-culture medium. Hence, all observed cellular responses would be related solely to the electrical stimulation (McCaig et al., 2005; Dubey et al., 2011). In a parallel and separate group of experiments, the viability (alamarBlue) of stimulated and control HUVECs was measured, to allow the construction of a temporal viability profile under stimulation. The impedance of cells was measured and analyzed over the range 10 mHz—1.0 MHz and over a 72 h period to monitor cell growth and proliferation formation of a confluent monolayer. Over similar periods, separately stimulated cells were isolated for total RNA extraction followed by gene expression via real time quantitative polymerase chain reaction (RTqPCR). The expression of downstream targets of the transcriptional coactivators, YAP/TAZ, were assessed by RTqPCR. Of specific interest was gene expression of connective tissue growth factor (CTGF) and ankyrin repeat domain 1 (ANKRD1) (downstream targets of YAP/TAZ activation) and VE-Cadherin (to assess endothelial junctional formation).

## Results

### Cell viability

**FIGURE 2:**
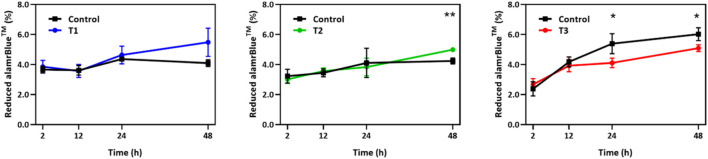
Temporal changes in alamarBlue cell viability assay over a 48 h period for HUVECs receiving electrical stimulation under T1, T2, and T3 electrical stimulation regimens. Percent of reduced alamarBlue shows the change in intensity relative to that obtained 1 h post cell seeding (*n* = 3). Statistically significant pair of data at *p*-values of <0.05 and <0.01 are indicated with * and **, respectively.

The alamarBlue cell viability assay was performed to monitor cell growth and any unfavorable side effect of EF on cells in comparison to a non-stimulated control group. As shown in Figure 2, during the first 24 h, the viability of the EF stimulated group followed the control group very closely under T1 and T2 EF stimulation regimens and outpaced the control thereafter. The average growth index after 48 h was 37.5% and 5% higher than control for T1 and T2 regimens, respectively. After 12 h under the T3 regimen, the growth index of the electro-stimulated group fell behind the control, with the average being 5% less than control after 48 h. Considering the ∼24 h doubling time of HUVECs, the data suggest that within two generations of cells, EF stimulation supported increased proliferation in a field dependent fashion. The data further suggest that the EF stimulation favorably altered HUVEC viability after 24 h under T1, after 48 h under T2 regimens, and unfavorably after 12 h under T3 EF regimen. Under T1 and T3 regimens, the influence of the EF on HUVEC viability was noticeable within the first generation of cells, 24 h. Cell viability was investigated beyond 48 h, including 72 h and 96 h. However, HUVEC viability was the same as the control beyond 48 h indicating that the electric field influence manifest in viability differences only within the first 48 h. The viability influences may relate to the cell cycle and suggests an adaptive response experienced by the cells.

### TEER measurement

**FIGURE 3:**
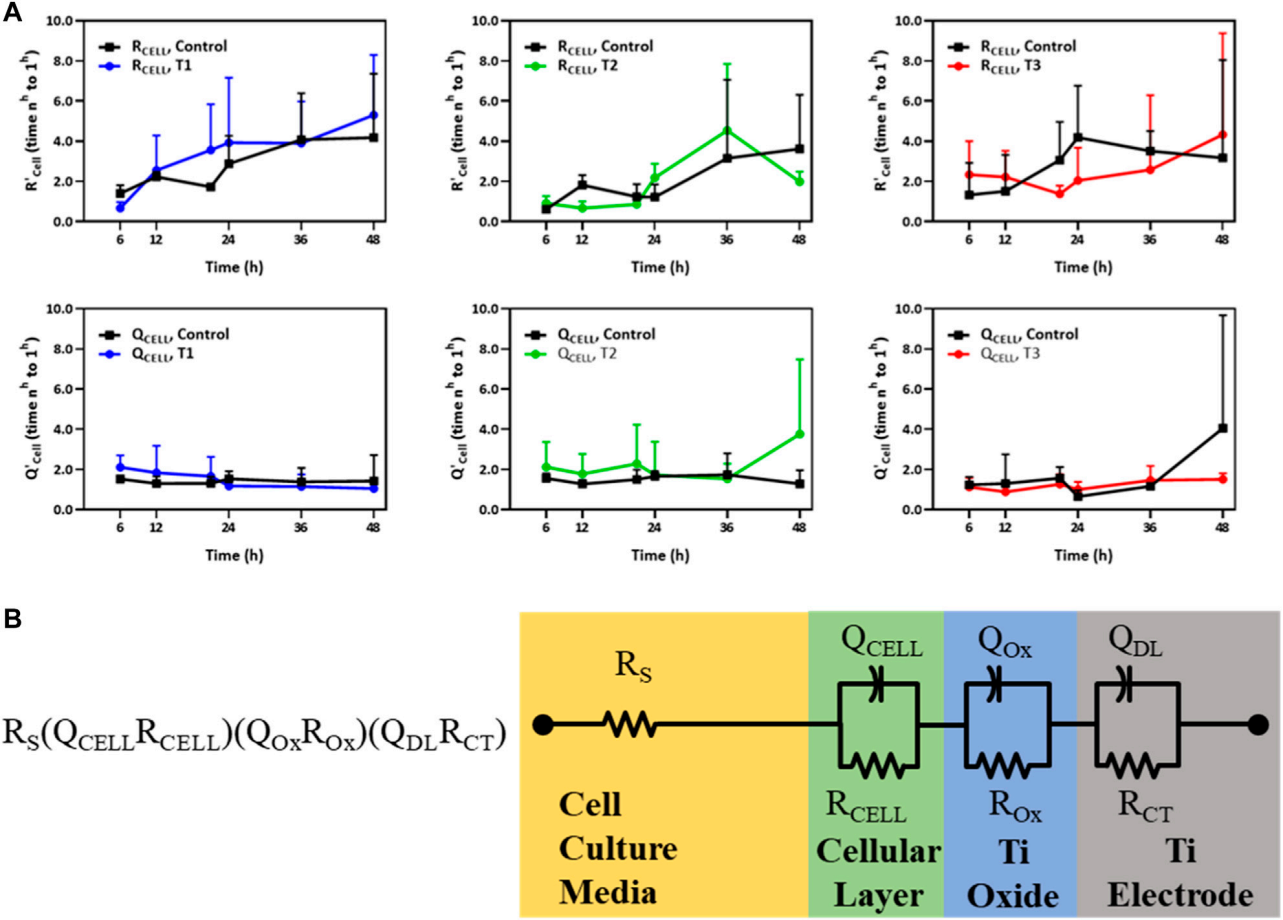
**(A)** Temporal changes of R’_CELL_ and Q’_CELL_ derived from the R_S_ (Q_CELL_R_CELL_) (Q_Ox_R_Ox_) (Q_DL_R_CT_) model for HUVECs electrostimulated by electric fields under the three different test regimens (T1, T2 and T3). The *Y*-axis in the top and bottom panels represents the relative fold change in R’_CELL_ (top panel) and Q’_CELL_ (bottom panel), respectively, normalized to their respective values measured 1 h post cell seeding (*n* = 3). **(B)** An equivalent circuit schematic of the R_S_ (Q_CELL_R_CELL_) (Q_Ox_R_Ox_) (Q_DL_R_CT_) model for the Titanium (Ti) electrode and its association with the cellular layer and the cell culture media EIS-EQCRT model parameters [Adapted from Abasi et al. (2020b)].

The trans-monolayer resistance of HUVECs was investigated via MBEIS using the concomitant recording mode of the ECSARA and characterized using EQCRTA with an appropriate model to describe the system. As in previous work, use was made of an R_S_ (Q_CELL_R_CELL_) (Q_OX_R_OX_) (Q_DL_R_CT_) equivalent circuit wherein R_S_ represents the resistance of the solution or medium, R_CELL_ and Q_CELL_ represent the insert-supported cell monolayer with R_CELL_ specifically being reflective of TEER, a measure of tight junction formation between HUVECs as illustrated in Figure 3B. Due to the non-ideal capacitive behavior of the bio-electrochemical system, the capacitance (C) is represented by a constant phase element (CPE or Q). The impedance of the CPE [Q=(1/C) (jω)^−n^], where for the case of n = 1, Q presents an ideal capacitor. The subscript “OX” stands for oxide layer and characterizes the electrochemical contribution of the titanium oxide layer formed on titanium electrodes. The Q_DL_ and R_CT_ terms reflect the double layer (DL) capacitance and charge transfer (CT) resistance in the electrochemical system, respectively. The temporal changes in Q_CELL_ and R_CELL_ correspond to the growing influence of tight junctions formed between the adjacent HUVECs as they grew to confluency. Values of Q_CELL_ and R_CELL_ were extracted and graphed and are shown in Figure 3A. In the measurement of TEER, R_CELL_ was observed, in the case of the T1 regimen, to outpace the control after 12 h, and in the case of the T2 regimen, R_CELL_ was observed to outpace the control after 24 h, consistent with the cell viability study. However, in the case of the T3 regimen, R_CELL_ was never observed to outpace the control. A similar pattern was observed in Q_CELL_, an element representing the number of cells in the EIS. Under the T3 regimen, the pattern was reversed and both R_CELL_ and Q_CELL_ were lower than control during the later phase of culture, again, consistent with and confirming the association of TEER with the viability study.

### Immunofluorescence of YAP localization

**FIGURE 4:**
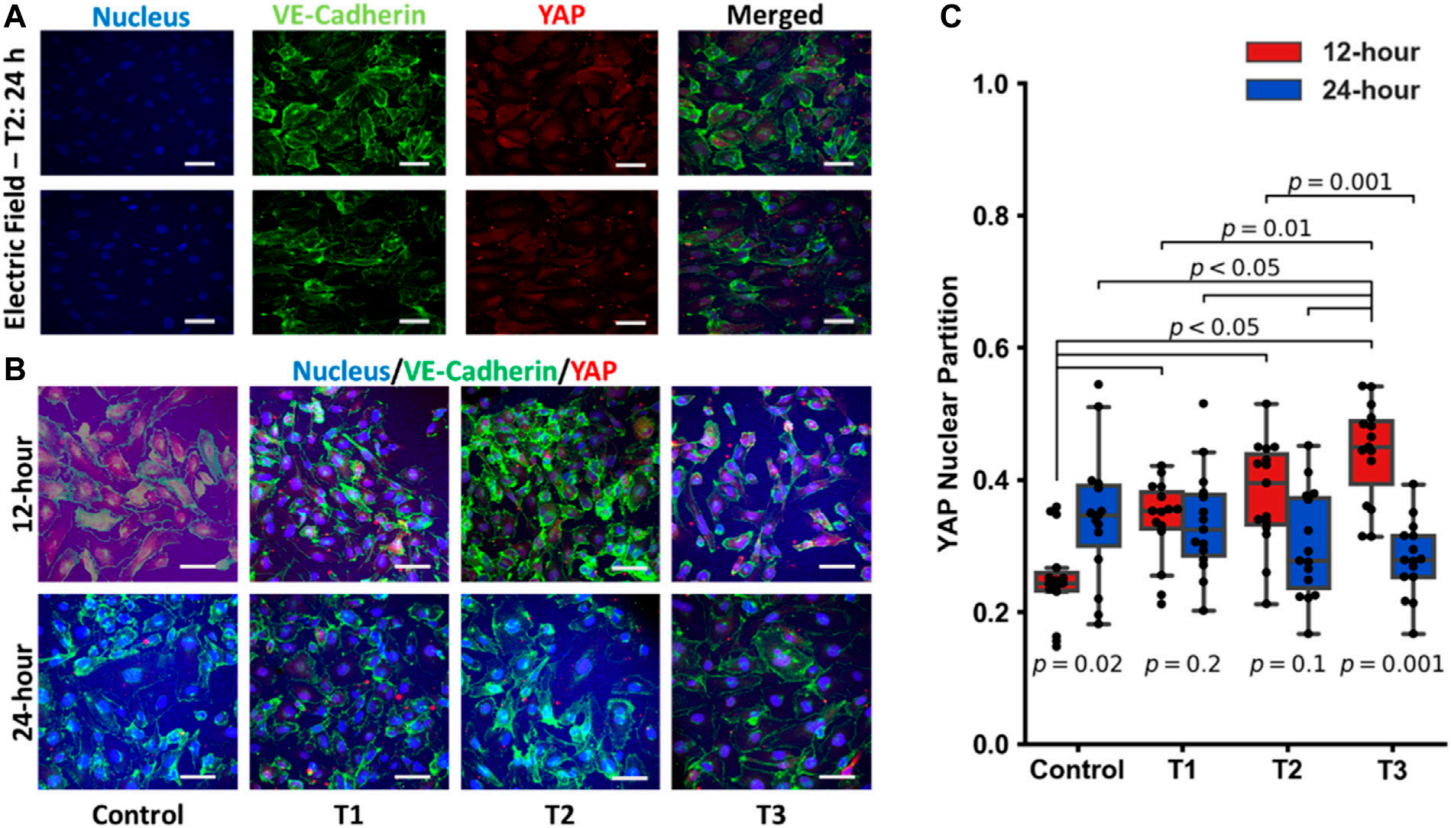
**(A)** Individual images of immunostaining of the nucleus, VE-Cadherin, YAP and the merged images for HUVECS that were electrostimulated by electric fields under the T2 test regimen after 24 h (scale bar = 50 µm). **(B)** Merged images of immunostaining of pre-confluent layers of HUVECs stimulated by electric fields under the three different test conditions (T1, T2 and T3) at 12 h and 24 h post stimulation (scale bar = 50 µm). **(C)** Box and Whisker plots of YAP nuclear localization obtained from immunofluorescent images, *n* = 15 individual cells per group. Each dot on the box plot represents an individual cell’s ratio of the nuclear signal to total cellular signal at 12 h (red) and 24 h (blue) post stimulation.

Previous reports have suggested that electric fields promote vascular proliferation and tight junction formation (Zhao et al., 2004; Li et al., 2017; Geng et al., 2019; Abasi et al., 2020a). Effectors of the endothelial cell mechanoresponse, YAP and TAZ (Dupont et al., 2011; Hong and Guan, 2012; Aragona et al., 2013; Piccolo et al., 2014; Wang K. C. et al., 2016; Wang L. et al., 2016), are known to modulate the cell cycle and control cellular proliferation and apoptosis. YAP/TAZ displays differential activity depending on the physical and biochemical stimuli and translocate to the nucleus when transcriptionally active. To explore how electrical fields may modulate these transcriptional co-activators, YAP/TAZ localization was assessed via immunostaining and subsequent confocal microscopy detailed in Figure 4. The use of RFP-HUVECs and staining with rabbit anti-YAP and secondary staining with donkey anti-rabbit Alexa Fluor™ 555 necessitated that background fluorescence be accommodated. This was done by reporting only relative emission changes expressed as the ratio of nuclear emission to total emission, any change being due to YAP localization. Each dot on the box plot in Figure 4B represents a single cell, with each partition value reflecting the ratio of nuclear YAP to total YAP, which is one metric by which relative levels of activity can be assessed. Increasing levels of this ratio reflect elevated nuclear YAP levels and, subsequently, endothelial cell activation (Wang L. et al., 2016). YAP/TAZ partitioning was most significant temporally, with time under electrostimulation being the statistically significant factor (*p*-values displayed on Figure 4B). Across the first 12 h of the three electro-stimulations regimens, YAP/TAZ nuclear partition was lowest in the control group and increased in all groups following electrical stimulation for 12-h. That is, electrostimulation of any type produced statistically significant YAP/TAZ partitioning (*p* < 0.05) following 12 h of electrostimulation, regardless of the magnitude and/or frequency of the stimulation (Figure 4B red blocks). At the 12 h time point, cells under T3 displayed the most elevated nuclear partition (*p* < 0.05), suggesting the T3 regimen was a potent inducer of YAP activity and proliferation; however, the cell viability data suggests otherwise, which may be tied to countervailing effects associated with elevated YAP activity (such as inflammation). When cells were again measured at 24 h, YAP/TAZ activity was not significantly different across all three regimens and the control, with the control showing significant elevation (Figure 4B blue blocks), implying that the cellular monolayers had reached comparable steady states after two generations, clearly demonstrating a temporal adaptive response. Immunofluorescence studies revealed that YAP/TAZ partitioning under these electrostimulation conditions should be viewed holistically in time and not for its influence by the specific magnitude or frequency of electrostimulation. The first is that in the 12 h group (where cells were actively proliferating) all electrically stimulated groups had an upshift in the YAP nuclear partition. After 24 h, this differential disappeared, and each group was found to be statistically indistinguishable, reflective of temporal adaptation to the stimulation conditions.

### Gene expression

**FIGURE 5:**
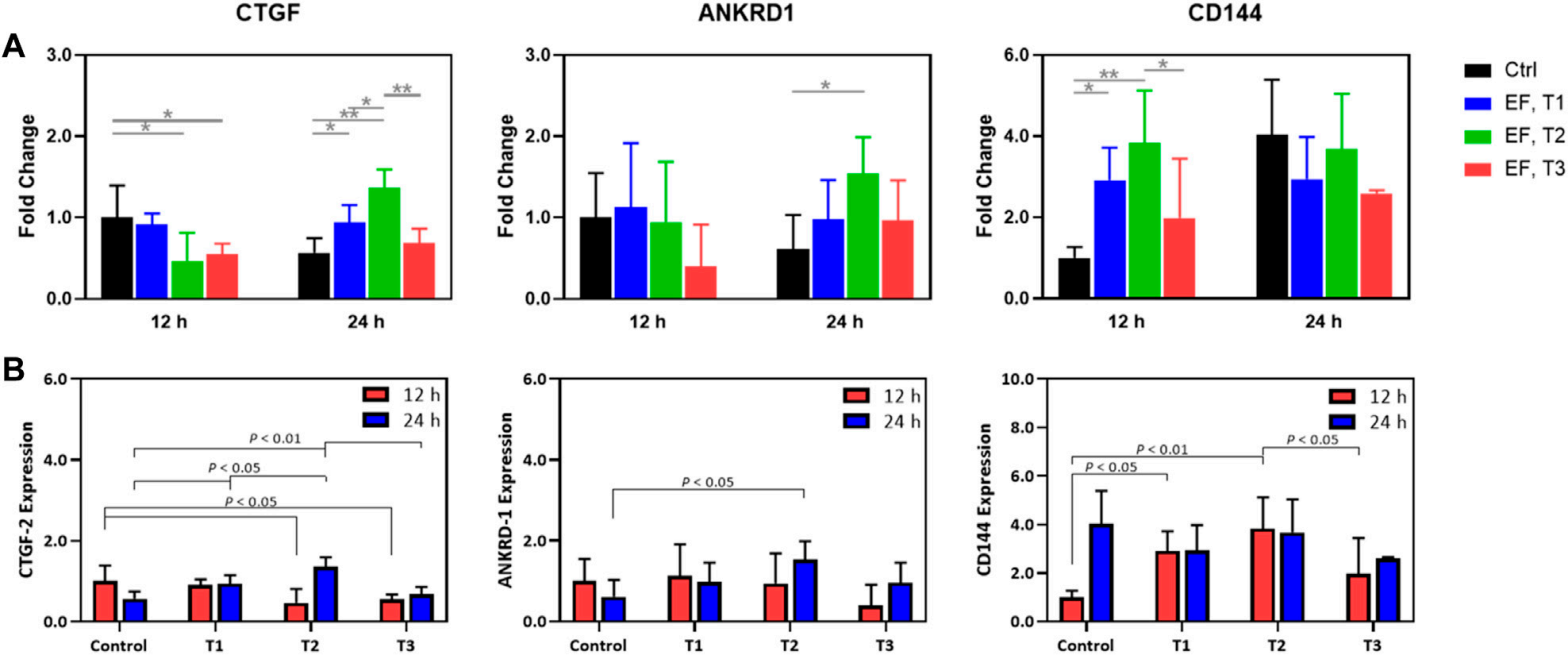
**(A)** Expression fold change relative to that of controls (unity) of CTGF-2, ANKRD-1, and CD144 in HUVECs that were electrostimulated by electric fields under the three different test regimens (T1, T2 and T3). **(B)** Expression fold change normalized to the control of CTGF-2, ANKRD-1, and CD144 in HUVECs that were electrostimulated by electric fields under the three different test regimens (T1, T2 and T3) and shown to emphasize the statistically significant differences among the test regimens at 12 h (red) and 24 h (blue) post stimulation. Results were obtained from RT-qPCR analysis (*n* = 3).

Next, specific targets of YAP/TAZ transcriptional activity (CTGF and ANKRD1) and expression of an endothelial junctional protein (VE-Cadherin/CD144) were measured in time and under EF stimulation (Figure 5). CTGF expression at 12 h was modulated modestly, with the T2 and T3 regimens producing a decrease in CTGF expression. At the 24 h mark, there was some upregulation through the different electrical stimulation regimens, although T3 was not different from the control 24 h group. Notably, the fold changes for each of these experimental groups rarely exceeded 2-fold but were always significantly increased relative to background. This contrasts with the IF data, as the IF data demonstrated an increase in YAP nuclear localization whereas gene expression showed a small downregulation. This might be tied to specific system limitations. ANKRD1 was not appreciably modulated by electrical stimulation across regimens with an exception being between the 24 h control and the T2 stimulation regimen at 24 h. In summary, for YAP targets, these genes do show some modulation, although the expression changes are less than 2-fold. In contrast, CD144 was dramatically upregulated by the T1 and T2 regimens, although this effect was significantly dampened by the T3 regimen as illustrated in the IF images of pre-confluent layers of HUVECs. We note that T3 was shown to function as more of an inhibitor of cell proliferation in the viability assays, which is captured here as well. At 24 h, there were no further changes in CD144 expression, although expression remained elevated compared to the 12 h control. This supports the view that certain electrical stimulation patterns can accelerate cell proliferation and junctional formation. After a certain time, cells proliferate to a steady state, agnostic of the electrical stimulation regime. Thus, the feasibility of electrically stimulating HUVEC proliferation and junctional formation is consistent with our previous purely cellular report (Abasi et al., 2020a).

## Discussion

This study was inspired by investigating potential homology between electro- and mechano-biology. In response to environmental changes, activation of upstream channels followed by intracellular signaling determines the cell’s response to a stimulus. Flow sensitive potassium and chloride ion channels are activated in response to shear forces causing cells to hyperpolarize/depolarize. Potassium ion channels demonstrate the first and fastest response to the shear stress, activating at lower thresholds and saturating at high stresses (Cooke et al., 1991; Ohno et al., 1993). These channels temporally lose their sensitivity under sustained stress (Olesen et al., 1988). Similar forces result in YAP/TAZ translocation between the cytoplasm to the nucleus. The response to steady and oscillatory shear stress is very different in endothelial cells according to several reports (Nuccitelli, 1988; Lieu et al., 2004; Wang K. C. et al., 2016; Wang L. et al., 2016; Dominic et al., 2020; Walther et al., 2021). Oscillatory flow of 0.2 Hz resulted in full hyperpolarization (2.8 mV) and limited depolarization (1.1 mV) while oscillatory flow of 5.0 Hz induced neither hyperpolarization nor depolarization. Oscillatory flow of 1.0 Hz caused partial hyperpolarization (1.8 mV) and depolarization (0.6–1.1 mV) (Lieu et al., 2004). In the case of steady shear stress, the threshold magnitude is much lower for hyperpolarization compared to depolarization. The same may be true of oscillatory electric fields of appropriate magnitude. It is arguable that electric fields exert an electromotive force on cells that parallels mechanical forces such as shear.

In the present study, a modest electrophysiologically relevant potential difference of 1.2 V or 0.6 V corresponding to an electric field of 162 mV/mm or 81 mV/mm was applied to HUVECs. This low electric field is within the physiological EFs (1–200 mV/mm) present in the body (Nuccitelli, 1988). Applying a perpendicular EF of this range to an approximate 2 µm thick endothelial cell monolayer (Ho et al., 2017), results in a potential difference of 0.32 mV or 0.16 mV across cells. Elucidating the exact nature of these mechanisms is an active area of research (Thrivikraman et al., 2018). These low potential differences could affect, and possibly activate, some of the voltage-sensitive channels of the cell membrane and may trigger signals to induce YAP/TAZ activation via a known mechanobiological pathway (Pathak et al., 2014; Thrivikraman et al., 2018). Such potentials also likely cause electrophoretic movement of ions (Ca^2+^, K^+^, Cl^−^, Na^+^) within the cell and cell culture media, creating a local, transient imbalance of ions across the cell membrane yet to affect transmembrane channels. And lastly, there may be some level of electromechanical coupling present as several surface receptors have a charge and would thus experience a force when subject to an external EF (Hart, 2006). Electromechanical coupling that induces force transduction via charged surface integrin receptors was proposed by Hart in 2006 (Hart, 2006), who postulated that an alternating EF of 100 mV/mm exerts forces of 1 fN on integrin molecules, similar to that of an oscillatory shear stress of 1 N/m^2^ (Hart, 2006). This force is independent of frequency up to a threshold before rapidly attenuating with increasing frequencies. The threshold frequency depends on the length of the charged glycoprotein, and is roughly within 0.1–1.0 Hz for glycocalyx on endothelial cells (Hart, 2008). Surface integrins are implicated in several mechanotransduction pathways and modulating their activity via external EFs may connect the electric stimuli with charged transmembrane molecules that transduce mechanical forces to the actin cytoskeleton. The coupling between glycoproteins on the surface of the same cell or adjacent cells signifies the efficiency and strength of the effect. More recently, experimental evidence for direct influence of charged residues of proteins under an electric field was demonstrated (Hekstra et al., 2016), providing additional experimental evidence that electromechanical coupling is an important contributing mechanism. Nevertheless, the EFs regimens employed were not sufficient to generate heat and/or change the local pH and/or ion concentration within the media (McCaig et al., 1994).

The engineered approach discussed here mainly focuses on demonstrating a systematic approach to integrating the relevant physical stimulus into a conventional cell culture system enabled by a high throughput approach to explore how electric fields affect molecular pathways. As shown in previous work (Abasi et al., 2020a) and this present report, a main advantage of this approach is isolation of the electric field from other effects conventional cell culture approaches have on cell behavior. Conventionally, HUVECs are cultured on stiff, polystyrene/borosilicate glass cell cultureware dishes/plates, which are known to support cellular proliferation and spreading. Using fashioned substrates of polydimethylsiloxane (PDMS) of controllable stiffness (∼800 kPa–10 MPa) to mimic the mechanical properties of the extracellular matrix, it was found that softer substrates attenuate proliferation and promote cell consolidation and tissue formation (Lo et al., 2000; Guo et al., 2006). Photolithographically fabricated microfluidic devices have allowed the study of variable flow rates on HUVECs, much like the endothelium experiences within the vasculature. When variable substrate stiffness is combined with variable flow rates new biological insights are revealed and these are centered on YAP (Walther et al., 2021). Here, another factor, electric field, is explored and the effects of EFs on cell proliferation are isolated from common technique effects to observe an increased proliferation rates and effect on YAP biology and CD144 expression in relation to TEER. The trans-monolayer resistance and capacitance are derivative properties that arise from the establishment of tight junctions between and among the HUVECs within the plane of cell growth. Setups that have been commonly used to study the influence of electric fields on cells place electrodes in direct contact with cells confounding the results with uncontrolled redox events (Thrivikraman et al., 2018) or place cells in the plane of a horizontal E-field such that all cells do not experience the same field strength; here, through the engineered approach, the work is more directly comparable to findings within the biological literature on physical stimuli and cell proliferation (Aragona et al., 2013). The benefit is a capacitive, non-invasive stimuli for studying electrobiology (Thrivikraman et al., 2018) to explore cell behavior using methods more closely approaching the complexity of living organisms (Tyler, 2017).

The voltage applied to the cells is negative relative to the ground plane (0 V) (Figure 1B). During pulse stimulation the electric field is reversed, and a positive voltage is applied to the cells relative to the ground plane (0 V) for a period corresponding to the duty cycle. The applied voltage is symmetrical relative to the zero plane. The constant potential applied to cells in our experiments likely polarized the cell monolayer and rearranged the charge distribution across the cells. The results of viability, where the number of live cells in T1 and T2 EF stimulation regimens but not that of T3, were higher than the control condition, confirmed that the T1 and T2 regimens promoted cell viability and/or proliferation while T3 suppressed the growth or caused cell death. These results were similarly reflected in EIS derived TEER data. In T1 and T2 regimens, the EF was applied at the same 1.2 Hz frequency with 0.6 V and 1.2 V pulse magnitude, respectively; while under the T3 regimen, a 0.6 Hz frequency with 1.2 V pulse was used. These results highlight the more determinative role of frequency (within this low frequency regimen) compared to the EF strength on cellular behavior. To test this visual conclusion, we normalized the three studied parameters, cell viability, EIS derived data (R_CELL_ and Q_CELL_), and gene expression (GTFC, ANKRD1, and CD144) at time points 12 h and 24 h for each regimen to the control. Using JMP, the normalized data were fit to a linear regression model with frequency and amplitude as effects. The model showed that at time 12 h, the response was independent of neither amplitude nor frequency; however, the response at 24 h was dependent on frequency with *p*-value = 0.034. The effect of amplitude on responses was not statistically significant confirming the importance of frequency in electrobiology. During the first 12 h, the applied electric field did not cause any difference in HUVEC population as inferred from viability assay, yet YAP translocated and partitioned into the nucleus at a significantly higher level under all three electro-stimulation regimens compared to the control. Alexa Fluor 555 staining of YAP for localization occurs against a background of RFP emission of the RFP-HUVECs. Cell-to-cell or sub-cellular regional RFP background variability was accommodated by ratioed measurement over many cells (*n* = 15). The results of immunofluorescent staining confirmed that across all three electro-stimulation regimens, electrical stimulation for 12 h increased YAP nuclear partitioning compared to the controls, while after 24 h, there were no longer significant differences. YAP/TAZ activity controls cellular proliferation and is supported by the general upshifting of CD144 expression in stimulated HUVECs, however, neither the viability data nor the gene expression data appear to follow this phenomenon on the same time scale. Notably, YAP/TAZ partitioning occurs at a timescale significantly faster than the target gene expression (minutes vs. hours) (Zhao et al., 2012), while the experiments here had a minimum time resolution of 12 h, thus explaining these disparities. This reinforces the well-established paradigm that subcellular signaling events occur on time scales that are far shorter than changes in morphological or pathological cellular and tissue processes, in this case viability and tight junction formation among HUVECs (Díaz-Coránguez et al., 2019). The effect of EF on cell population was observed 12–24 h post stimulation, in accord with the cell doubling time of ∼24 h. However, at the 24-h mark, nuclear partitioning does not display significant differences indicating that at the 24-h mark, the endothelial monolayer has reached confluency, and thus is biologically more quiescent.

Gene expression of downstream targets of YAP was modestly regulated in the electroculture system, and in some cases were opposite to that supported by cell viability assays and IF studies. This may be due to several reasons tied to the system itself, as the culture wells are relatively non-physiologic physical environments which interferes with YAP/TAZ activity. Such outcome, however, are not likely to be related to pH/temperature changes caused by the electric field since the field strengths applied were generally too weak to split water or cause Joule heating. Other parameters which modulate YAP activity strongly, such as substrate stiffness (Dupont et al., 2011; Aragona et al., 2013) and shear stress, although controlled (no flow employed and the same substrate used), were not directly measured. Gene expression is notably convoluted, with specific variables known to influence downstream YAP activity, which explains the relatively low fold-change observed with CTGF, as its expression is extremely sensitive to YAP activity. Conversely, ANKRD1 has several other regulatory pathways and is not as strongly influenced by YAP activity as is CTGF (Wang L. et al., 2016). However, expression of VE-Cadherin was clearly accelerated in the presence of electric fields, consistent with previous reports demonstrating that electrical stimulation increased cell proliferation and junctional formation (Zhao et al., 2004; Geng et al., 2019). The 12 h time points associated with cellular proliferation and junctional formation in the viability and impedance studies all showed stark upregulation on qPCR, while the group that inhibited growth (T3) showed diminished CD144 expression compared to the T2 group which promoted expression most strongly. At 24 h, cells achieved a steady state, and CD144 expression stabilized across the three regimens regardless of the stimulation magnitude and pattern. Outside of the system limitations themselves, each experiment was conducted in the same test environment (notwithstanding the applied electric field) alongside an un-stimulated negative control group, so changes that are observed are attributable to the electrical stimuli. Together then, the data presented here is consistent with previous reports, yielding a closed system which allows for control over the electrical stimulation regimes in a precise manner. The impact produced by the quite modest E-fields, inspired by voltages and frequencies found in endogenous biology, are highly nuanced and their impact with statistical significance were revealed only through the large data sets enabled by multiplexed 24-well experiments. While there is desire to seek optimal electrostimulation conditions, to identify threshold values of frequencies and voltages that affect particular levels of gene expression or cellular responses, and to conduct RNA sequence analysis, these are outside the scope of this paper.

Electric fields exert an electromotive force on cells that parallels mechanical forces such as shear. Accordingly, like the action of shear on HUVECs, YAP localization is enhanced because of electrostimulation. However, this is apparent only in the short time scale, 12 h. Downstream expression, associated with YAP localization, is not so clearly defined suggesting that while there may be parallels, there may also be differences in the detailed mechanism of action. Non-etheless, this report goes beyond others to confirm that electric fields share a similar influence as do mechanical forces in affecting YAP nuclear partitioning. Electric fields also appear to promote CD144 expression, noted for its role in tight junction formation.

Future work in this area may include the design and application of microfluidic systems with appropriately modified luminal electrodes (Rapier et al., 2022) for the simultaneous application of shear stresses, electrical stimulation, and NO sensing. Additional future work in this area may include a test of the hypothesis that EF stimulation triggers the same or similar pathways as mechanical shear stress. Such experiments may be threshold gating based on the additive contributions of shear and EF, may involve genetic knockouts, or the use of pharmaceuticals such as verteporfin, which binds to YAP and inhibits its nuclear localization or Y-27632, which inhibits the Rho-associated protein kinase (ROCK) pathway and promotes YAP/TAZ nuclear localization and activity. Other noteworthy compounds include the statins, which inhibit HMG-CoA reductase and activate the Hippo pathway, and mevalonate, which can activate YAP/TAZ by increasing the levels of geranylgeranyl pyrophosphate (GGPP), a lipid molecule that is required for YAP/TAZ activity.

## Conclusion

The ECSARA system enabled high throughput experiments for the study of EF effects on gene expression in HUVECs–a path toward understanding electro-stimulated vascularization. It was found that, overall, EF stimulation expedited junctional formation, increased cellular proliferation, and increased YAP nuclear localization, linking electrobiology and mechanobiology via electromics. The YAP localization, which is well known to be a signature of mechano-stimulation in mechanobiology, is likewise shown to be responsive to electrical stimulation. There is thus a possible parallel between one aspect of the mechanobiology response and the electrobiology response–YAP/TAZ localization. However, downstream targets of YAP were not appreciably affected, likely due to countervailing physical stimuli that are also known to govern YAP activity (such as substrate stiffness), which, being on nano-porous transwell insert surfaces, were not controlled in a manner to avoid potentiation. Regardless, the results demonstrate that electrical stimulations improve proliferative capacity, and that oscillatory frequency was more significant than field strength. This has several implications in regenerative medicine, with potential applications in wound healing and in tissue regeneration, especially in the context of revascularization of tissues to restore blood flow. Together, these data support that EFs modulate gene expression tangibly which may see application in the clinic as electroceutical treatments in regenerative medicine. This work opens the field of electromics for further investigation, or more precisely, quantifying the effect of electric fields on gene expression and cell biology to understand how this transduction occurs and the biochemical pathways that are modulated. Future work includes a holistic approach using sequencing methods to provide a global look into gene expression under electrical influence.

## Materials and methods

### Electrical stimulation of HUVECs

RFP-expressing HUVECs (Angio_Proteomie) were cultured and expanded according to standard protocols (Marin et al., 2001). Cells at passage number ≤8 and hence doubling times ∼24 h were used for all experiments. Following expansion, cells were seeded on gelatin-coated (2 wt% in PBS 7.4 for 1 h at 37°C) 0.4 µm pore size PET (polyethylene terephthalate) inserts (Corning, Millipore) at a density of 2.5 × 10^5^ cells/ml (equal to 7.5 × 10^4^ cells/cm^2^) which were then placed in ECSARA. Several seeding levels were explored before arising at a seeding density of 2.5 × 10^5^ cells/ml (equal to 7.5 × 10^4^ cells/cm^2^). ECSARA is an electrically enabled cell stimulation and recording apparatus designed and developed at the C3B Labs. It is a 24-well cell culture plate equipped with a vertically arranged opposing pair of titanium electrodes, one in the top plate and the other in the bottom plate of each well. This creates a uniform electric field orthogonal to the cell growth plane of the insert while enabling their electrical stimulation and the measurement of their electrical impedance. The design, fabrication, modeling, testing, and validation of the system has been previously reported (Abasi et al., 2020a). One hour post seeding, t_0_, the TEER was measured at an interrogation voltage of 20 mV p-p over the frequency range of 0.01 Hz–1 MHz.

Electrostimulation of cells was initiated immediately following TEER (t_0_) measurement, which was 1 h post seeding. Three electrical stimulation regimens (T1, T2 and T3) were evaluated and each regimen was evaluated temporally for 12 h, 24 h and 48 h. An electrical pulse of T1 = 0.6 V magnitude (81 mV/mm), 2 mS width and 1.2 Hz; T2 = 1.2 V magnitude (162 mV/mm), 2 mS width and 1.2 Hz; and T3 = 1.2 V magnitude (162 mV/mm), 2 mS width and 0.6 Hz as shown in Figure 1. The frequency of 1.2 Hz was selected for its analogy to the typical heart rate of 72 bpm. Stimulation was applied continuously to cells except for 30 min interval when the EIS-TEER was measured. Results were compared with cells that were simultaneously and similarly cultured at electric fields of 0 (non-stimulated control). The EIS data were collected from the cells every 6 h in the first 24 h and then every 12 h and were modeled using an R_S_ (Q_CELL_R_CELL_) (Q_OX_R_OX_) (Q_DL_R_CT_) equivalent circuit wherein R_S_ represents the resistance of the solution or medium, R_CELL_ and Q_CELL_ represent the insert-supported cell monolayer with R_CELL_ specifically being reflective of transmembrane epithelial/endothelial cell resistance (TEER), a measure of tight junction formation among HUVECs.

### HUVEC viability

The viability of controls and of EF-stimulated HUVECs were measured using alamarBlue bioassay. Controls were measured at 1 h post seeding (t_0_) and prior to any hybrid electrostimulation-TEER experiments. To establish cell viability following the completion of hybrid electrostimulation-TEER experiments (T1, T2 or T3) of duration 12 h, 24 h or 48 h, a new t_0_ was established for a triplicate group of wells for which the usual cell culture media was replaced with media containing 10% alamarBlue reagent. For both controls and test regimens, cells were incubated for 2 h (EF off) and the absorbance was subsequently measured with a Synergy HT plate reader (BioteK) at 570 nm and 600 nm wavelengths.

### cDNA synthesis and gene expression

RNA was extracted using the Quick-RNA Miniprep Kit (ZYMO Research Inc.) and the protocol recommended by the manufacturer was followed step-by-step. Extracted RNA was converted to cDNA in a 20 µL reaction using 5x iScript Reverse Transcriptase Supermix (Bio-Rad Laboratories, Inc.). The reaction volume was then diluted to 100 µL using DNAse/RNAse-free water. For each qPCR reaction, a volume of 10 µL was used consisting of 5 µL of Power SYBR Green Master Mix (Applied Biosystems, Thermo Fisher Scientific), 3.5 µL of H_2_O (DNAse/RNAse free, molecular grade), 0.5 µL of the gene specific primer, and 1 µL of the sample cDNA. All primers were purchased as validated 20x SYBR Green assays for qPCR (Bio-Rad Laboratories, Inc.). Primers used were glutaraldehyde 3-phosphate dehydrogenase (GAPDH), connective tissue growth factor (CTGF), ankyrin repeat domain 1 (ANKRD1), and VE-Cadherin (CD144). RT-qPCR was performed using a QuantStudio 12K Flex (Applied Biosystems, Life Technologies) with the pre-set settings of: MicroAmp EnduraPlate Optical 384-well plate (Applied Biosystems, Thermo Fisher Scientific), Relative Quantification (-ΔΔCt), SYBR Green Reporter, and Standard Run Time. Statistics were performed on ΔCt values. All gene expression results were reported as a fold change with standard deviation with respect to the denoted control and the housekeeping gene, Glyceraldehyde-3-Phosphate Dehydrogenase (GAPDH).

### Immunofluorescence

Each insert was placed into a new 24-well plate, washed with PBS and fixed using 4% paraformaldehyde (Thermo Fisher Scientific) for 20 min at room temperature. Cells were washed afterwards twice with PBS and then blocked and permeabilized with 2% bovine serum albumin (BSA, Sigma Aldrich) and 0.1% Triton X-100 (Thermo Fisher Scientific) in PBS (blocking buffer) for 1 h at 4°C. Each insert was then stained with rabbit anti-YAP (1:100) in blocking buffer overnight (16 h) at 4°C. The next day, inserts were washed twice with PBS and secondary staining was performed with donkey anti-rabbit Alexa Fluor 555 (Invitrogen Molecular Probes, Thermo Fisher Scientific) in blocking buffer for 1 h at room temperature. Actin was stained with phalloidin Alexa Fluor 488 (Invitrogen Molecular Probes, Thermo Fisher Scientific) in PBS for 30 min. Each trans-well insert was then carefully cut out to be mounted on a glass microscope slide. Nuclei were stained with 4′,6′-diamidine-2′- phenylindole dihydrochloride (DAPI, Roche Diagnostics), contained within the coverslip mounting media.

### Imaging and imaging analysis

Slides were imaged at 40x oil immersion (ULSAPO40XS NA: 1.25, Airy Disk: 1) using a FLUOVIEW FV3000 confocal microscope (Olympus Corporation). Cells were imaged directly on cutouts of the trans-well, nano-porous membrane, which affected image quality. Each condition was imaging using the Z-stack function (1.0 µm steps/10 slices per cell) and analysis was performed using maximum Z-projections on the associated software (cellSens, Olympus Corporation) for YAP partitioning. YAP partitioning was calculated as the nuclear YAP signal divided by the total cellular YAP signal using regions of interest manually created in the software for *n* = 15 cells.

### Data visualization

All data visualization was performed in Python 3.7 using Matplotlib (Hunter, 2007) with Seaborn packages or with GraphPad Prism. All Python code has been deposited in GitHub; example code is included in supplementary material.

## Data Availability

The raw data supporting the conclusion of this article will be made available by the authors, without undue reservation.
