# Supplementary material for: Electrically stimulated gene expression under exogenously applied electric fields
Source: Front Mol Biosci. 2023 May 4;10:1161191. doi: 10.3389/fmolb.2023.1161191 (PMC10192815; doi:10.3389/fmolb.2023.1161191)
Supplement: Supplementary file 1 [file Table1.pdf]

Supporting Information for

Electrically Stimulated Gene Expression under Exogenously Applied Electric Fields.

Sara Abasi[1], Abhishek Jain[1,2,3], John P. Cooke[2] and Anthony Guiseppi-Elie[1,2,4,5,*]

[1]Bioelectronics, Biosensors and Biochips (C3B), Department of Biomedical Engineering, Department of Electrical and Computer Engineering, Texas A&M University, College Station, TX 77843, USA

[2]Department of Cardiovascular Sciences, Houston Methodist Institute for Academic Medicine and Houston Methodist Research Institute, 6670 Bertner Ave., Houston, Texas 77030, USA.

[3]Department of Medical Physiology, College of Medicine, Texas A&M Health Science Center, Bryan, TX 77807, USA

[4]Division of Engineering and Industrial Technology, Tri-County Technical College, Pendleton, South Carolina 296701, USA

[5]ABTECH Scientific, Inc., Biotechnology Research Park, 800 East Leigh Street, Richmond, VA 23219, USA

*Anthony Guiseppi-Elie.

Email: guiseppi@tamu.edu

This PDF file includes:

Python 3.7 code using Matplotlib(1) with Seaborn packages or with GraphPad Prism.

Supporting Information Code

```
import seaborn as sns
import pandas as pd
import matplotlib
import matplotlib.pyplot as plt
import os
from matplotlib.patches import PathPatch
import numpy as np
from cycler import cycler

# Import data into pd datafram

path = "" # Input path here

os.chdir(path)
data = pd.read_excel(".xlsx")

#print(data)

# Global text/color parameters
```

```
#matplotlib.rcParams["font.weight"] = "bold"
matplotlib.rcParams["font.size"] = 10
matplotlib.rcParams["axes.linewidth"] = 1

# Set bar plot aspect ratio - X, Y
matplotlib.rcParams['figure.figsize'] = 3,4
matplotlib.rcParams['figure.dpi'] = 300

# Extra useful parameters
#matplotlib.rcParams['figure.dpi'] = 100
#plt.rc('axes', prop_cycle=(cycler('color', ['k', 'r', 'b'])))

colors1 = [#"black",
        "blue",
        "red"
        #"green",
        #"white",
        ]

# colors2 = [
#       "orange",
#       "green"
#       #"purple",
#       #"pink"]
#       ]

colors3 = ["black"]

# Create a box plot

f, ax = plt.subplots()

sns.set_style("ticks")

#Split plot
sns.boxplot(data=data, linewidth=1.5, palette=sns.xkcd_palette(colors1),
        hue="Time", x="Group", y="Index",
        width=0.8,
        showfliers=False)

sns.swarmplot(data=data, palette=sns.xkcd_palette(colors3),
        hue="Time", x="Group", y="Index",
        size=3,
        alpha=1,
        dodge=True,
        #zorder=1,
```

```
            clip_on=False)

####################################################
#                                                  #
# Box Plot Edge Color Adjustment                   #
#                                                  #
####################################################

box_patches = [patch for patch in ax.patches if type(patch) ==
matplotlib.patches.PathPatch]
if len(box_patches) == 0:  # in matplotlib older than 3.5, the boxes are stored in
ax2.artists
    box_patches = ax2.artists
num_patches = len(box_patches)
lines_per_boxplot = len(ax.lines)//num_patches
for i, patch in enumerate(box_patches):
    # Set the linecolor on the patch to the facecolor, and set the facecolor to None
    col = patch.get_facecolor()
    patch.set_edgecolor(col)
    patch.set_facecolor('None')

    # Each box has associated Line2D objects (to make the whiskers, fliers, etc.)
    # Loop over them here, and use the same color as above
    for line in ax.lines[i * lines_per_boxplot: (i + 1)*lines_per_boxplot]:
        line.set_color(col)
        line.set_mfc(col)  # facecolor of fliers
        line.set_mec(col)  # edgecolor of fliers

####################################################
#                                                  #
# Draw lines + Annotate                            #
#                                                  #
####################################################

#ax.set_ylabel("YAP Nuclear Partition", weight="bold")

ax.tick_params(width=1)

# Draw error bars and show statistics

# Statistical annotation
x1, x2, x3, x4 = 0, 1, 2, 3   # column indices
font_size = 8

# Paired Group Statistics
```

```
# # Group 1
# plt.text((x2), 0.05, r"$p = 0.007$", fontsize=8, ha='center', va='bottom', color="k",
clip_on=False)
#
# # Group 2
# plt.text((x3), 0.05, r"$p < 10^{-13}$", fontsize=8, ha='center', va='bottom', color="k",
clip_on=False)

# # Group 3
# plt.text((x3), 0.05, r"$p < 10^{-6}$", fontsize=8, ha='center', va='bottom', color="k",
clip_on=False)
#
# # Group 4
# plt.text((x4), 0.1, r"$p = 0.001$", fontsize="small", ha='center', va='bottom', color="k",
clip_on=False)


# Between Group Statistics
# These are various groups of bars that draw onto a figure directly. Some of the
values/annotations have been changed - the text function
# is what can be modified to alter what appears on the graph

# #Set Error Bar - Large Group 1
# y1, h1, h2, col1 = 0.41, 0.3, 0.27, 'k'
# plt.plot([x1+0.2, x1+0.2, x3+0.2, x3+0.2], [y1+0.05, y1+h1-0.04, y1+h1-0.04, y1+h2-
0.02], lw=1, c=col1) # Connecting Line (Bracket)
# plt.plot([x1+0.201, x2+0.2, x2+0.2], [y1+h1-0.08, y1+h1-0.08, y1+h2-0.06], lw=1,
c=col1) # Connecting Line (L)
# #plt.plot([x1-0.2, x2-0.2, x2-0.2], [y1+h1-0.08, y1+h1-0.08, y1+h2-0.06], lw=1,
c=col1) # Connecting Line (L)
# #plt.plot([x1+0.2, x1+0.2, x3-0.2, x3-0.2], [y1+h2, y1+h2, y1+h2, y1+h2], lw=1,
c=col1) # Group Line
# plt.text((x1+0.2+x3+0.2)*.5, y1+h1-0.04, r"$p < 10^{-6}$", fontsize=6, ha='center',
va='bottom', color=col1, clip_on=False) # Annotation
# plt.text((x1+0.2+x2+0.2)*.5, y1+h1-0.08, r"$p = 0.004$", fontsize=6, ha='center',
va='bottom', color=col1, clip_on=False) # Annotation

# #Set Error Bar - Large Group 2
# y1, h1, h2, col1 = 0.63, 0.3, 0.27, 'k'
# plt.plot([x1, x1, x2-0.2, x2-0.2], [y1+h2+0.001, y1+h2+0.02, y1+h2+0.02, y1+h2-0.12],
lw=1, c=col1) # Connecting Line (Bracket)
# # plt.plot([x4-0.2, x3+0.2, x3+0.2], [y1+h1-0.14, y1+h1-0.14, y1+h2-0.12], lw=1,
c=col1) # Connecting Line (L)
# # plt.plot([x4-0.2, x2+0.2, x2+0.2], [y1+h1-0.12, y1+h1-0.12, y1+h2-0.1], lw=1,
c=col1) # Connecting Line (L)
```

```
# plt.plot([x1-0.2, x1-0.2, x1+0.2, x1+0.2], [y1+h2, y1+h2, y1+h2, y1+h2], lw=1, c=col1)
# Group Line
# plt.text((x1+x2-0.2)*.5, y1+h2+0.02, r"$p < 10^{-10}$", fontsize=6, ha='center',
va='bottom', color=col1, clip_on=False) # Annotation

# # Set Error Bar
# y2, h2, col2 = 0.77, 0.015, 'k'
# plt.plot([x1-0.2, x2-0.2, x2-0.2], [y2+h2, y2+h2, y2], lw=1, c=col2)
# plt.text((x1-0.2+x2-0.2)*.5, y2+h2, r"$p < 10^{-12}$", ha='center', va='bottom',
color=col2, fontsize=6)
#
# # Set Error Bar
# y2, h2, col2 = 0.81, 0.015, 'k'
# plt.plot([x1-0.2, x1-0.2, x2+0.2, x2+0.2], [y2-0.24, y2+h2, y2+h2, y2], lw=1, c=col2)
# plt.text((x1-0.2+x2+0.2)*.5, y2+h2, r"$p = 0.002$", ha='center', va='bottom',
color=col2, fontsize=6)
#
# # Set Error Bar
# y2, h2, col2 = 0.68, 0.015, 'k'
# plt.plot([x1+0.2, x2-0.2, x2-0.2], [y2+h2, y2+h2, y2], lw=1, c=col2)
# plt.text((x1+0.2+x2-0.2)*.5, y2+h2, r"$p < 10^{-13}$", ha='center', va='bottom',
color=col2, fontsize=6)
#
# # Set Error Bar
# y2, h2, col2 = 0.72, 0.015, 'k'
# plt.plot([x1+0.2, x1+0.2, x2+0.2, x2+0.2], [y2-0.045, y2+h2, y2+h2, y2], lw=1, c=col2)
# plt.text((x1+0.2+x2+0.2)*.5, y2+h2, r"$p = 0.9$", ha='center', va='bottom', color=col2,
fontsize=6)

##############################################
# Draw single connecting bars (collapsed) #
##############################################

# # Set Error Bar
# y2, h2, col2 = 0.9, 0.015, 'k'
# plt.plot([x1, x1, x2-0.2, x2-0.2], [y2, y2+h2, y2+h2, y2-0.13], lw=1, c=col2)
# plt.plot([x1-0.2, x1+0.2], [y2-0.001, y2-0.001], lw=1, c=col2) # Draw a line
# plt.text((x1+x2-0.2)*.5, y2+h2, r"$p < 10^{-10}$", ha='center', va='bottom',
color=col2, fontsize=6)
#
# # Set Error Bar
# y2, h2, col2 = 1, 0.015, 'k'
# plt.plot([x1-0.2, x1-0.2, x2+0.2, x2+0.2], [y2, y2+h2, y2+h2, y2], lw=1, c=col2)
# plt.text((x1-0.2+x2+0.2)*.5, y2+h2, r"$p = 0.4 $", ha='center', va='bottom', color=col2,
fontsize=6)
#
```

```
# # Set Error Bar
# y2, h2, col2 = 0.95, 0.015, 'k'
# plt.plot([x1+0.2, x1+0.2, x2+0.2, x2+0.2], [y2, y2+h2, y2+h2, y2], lw=1, c=col2)
# plt.text((x1+0.2+x2+0.2)*.5, y2+h2, r"$p = 0.5$", ha='center', va='bottom', color=col2,
fontsize=6)
#
# # Set Error Bar
# y2, h2, col2 = 0.72, 0.01, 'k'
# plt.plot([x2+0.2, x2+0.2, x3+0.2, x3+0.2], [y2, y2+h2, y2+h2, y2], lw=1, c=col2)
# plt.text((x2+0.2+x3+0.2)*.5, y2+h2, r"$p = 0.3$", ha='center', va='bottom', color=col2,
fontsize=6)

# # Set Error Bar - Single
# y3, h3, col3 = 0.75, 0.01, 'k'
# plt.plot([x2-0.2, x2-0.2, x4-0.2, x4-0.2], [y3, y3+h3, y3+h3, y3], lw=1, c=col3)
# plt.text((x2-0.2+x4-0.2)*.5, y3+h3, r"$p = 0.01$", fontsize="small", ha='center',
va='bottom', color=col3)

# # Set Error Bar - Single
# y3, h3, col3 = 0.73, 0.01, 'k'
# plt.plot([x4-0.2, x4-0.2, x4+0.2, x4+0.2], [y3, y3+h3, y3+h3, y3], lw=1, c=col3)
# plt.text((x4-0.2+x4+0.2)*.5, y3+h3, r"$p = 0.001$", fontsize="small", ha='center',
va='bottom', color=col3)

sns.despine()

# Legend Parameters
handles, labels = ax.get_legend_handles_labels()
legend_properties = {'weight':'bold', "size":8}
ax.legend(handles=handles[:2], labels=labels[:2], prop=legend_properties,
        fontsize=9,
        frameon=False, bbox_to_anchor=(1.1, 1.05)
        #title_fontsize="small"
        )

# Also fix the legend
for legpatch in ax.get_legend().get_patches():
    col = legpatch.get_facecolor()
    legpatch.set_edgecolor(col)
    legpatch.set_facecolor("None")

plt.xticks(weight="normal")
plt.yticks(weight="normal")

ax.set(ylim=(0, 90))
ax.set(xlim=(-0.5, 3.5))
```

```
#plt.xticks(rotation=30)
plt.tight_layout()

ax.set_xticklabels(["CTRL", "RS", "CTRL", "HGPS"])

# Adjust Box Plot Width

####################################################
#                                 #
# Box Plot Spacing Adjustment               #
#                                 #
####################################################

def adjust_box_widths(g, fac):
    """
    Adjust the withs of a seaborn-generated boxplot.
    """

    # iterating through Axes instances
    for ax in g.axes:

        # iterating through axes artists:
        for c in ax.get_children():

            # searching for PathPatches
            if isinstance(c, PathPatch):
                # getting current width of box:
                p = c.get_path()
                verts = p.vertices
                verts_sub = verts[:-1]
                xmin = np.min(verts_sub[:, 0])
                xmax = np.max(verts_sub[:, 0])
                xmid = 0.5*(xmin+xmax)
                xhalf = 0.5*(xmax - xmin)

                # setting new width of box
                xmin_new = xmid-fac*xhalf
                xmax_new = xmid+fac*xhalf
                verts_sub[verts_sub[:, 0] == xmin, 0] = xmin_new
                verts_sub[verts_sub[:, 0] == xmax, 0] = xmax_new

                # setting new width of median line
                for l in ax.lines:
                    if np.all(l.get_xdata() == [xmin, xmax]):
                        l.set_xdata([xmin_new, xmax_new])
```

```
# Call Adjust Function
adjust_box_widths(f, 0.6)

plt.show()
```

References

1. J. D. Hunter, Matplotlib: A 2D Graphics Environment. *Computing in Science & Engineering* **9**, 90-95 (2007).
